# Identification of Immune-Active Peptides in Casein Hydrolysates and Its Transport Mechanism on a Caco-2 Monolayer

**DOI:** 10.3390/foods12020373

**Published:** 2023-01-13

**Authors:** Haiyan Xue, Jingjing Han, Jun Ma, Hongxin Song, Baoyuan He, Xiaofeng Liu, Meixia Yi, Lei Zhang

**Affiliations:** 1 School of Food Science and Engineering, Shaanxi University of Science and Technology, Xi’an 710021, China; 2 College of Bioresources Chemical & Materials Engineering, Shaanxi University of Science and Technology, Xi’an 710021, China

**Keywords:** casein, immune-active peptides, Caco-2 cells, polypeptide absorption mechanism, B-lymphocyte

## Abstract

In this study, we investigated the transport mechanism of immune-active peptide fragments isolated from casein gastrointestinal hydrolysates via a Caco-2 monolayer. The casein gastrointestinal hydrolysates could stimulate B-lymphocyte proliferation and reduce the TNF-α level. Then, we identified the bioactive peptide fragments derived from casein gastrointestinal hydrolysis using LC-MS/MS. Our results demonstrated that the transport mechanism of five immune-active peptides at the cell level was bypass transport. In addition, the majority of peptide RYPLGYL was transported through the monolayer cell membrane as an intact form for playing immune-active functions. The KHPIK and FFSDK were mainly degraded into small fragments, except for a small amount passing through Caco-2 cells in an entire form. Overall, these results suggested that casein or its immune-active peptides might play a role in regulation of the intestinal immune system.

## 1. Introduction

The protein content in milk is high, accounting for about 3.3–3.5% of the total mass, whereas casein accounts for 80% of the total protein in milk [1]. Casein is also the protein with the highest nutritional value in milk and one of the high-quality proteins for the human body [2]. After controlled *in vitro* enzymatic hydrolysis of casein, peptides with physiological activity were produced, such as immune-active peptides, antioxidant peptides, Angiotensin converting enzyme (ACE) inhibitory peptides, and casein phosphopeptides. Therefore, the physiological activity and function of casein after gastrointestinal hydrolysis need to be explored. Recently, it has been shown that some peptides can be absorbed into the circulatory system in a form without being decomposed into amino acids, providing a molecular biological basis for the research and application of immunocompetent peptides [3]. Immune activity evaluation methods are usually divided into *in vivo* and *in vitro* experiments. The *in vivo* experimental results are more reliable for complex environments, similar to human beings. However, because of the heavy workload, long experimental periods, and the limitation of individual differences in experimental models, *in vivo* methods are the mainly approaches for experiments, compared to *in vitro* [4]. Bohn and collaborators reported that the bioactive peptides released *in vitro* simulated gastrointestinal digestion were similar to that in *in vivo* experiments [5]. Therefore, managing *in vitro* conditions, including temperature, pH, hydrolysis time, appropriate protease, or combining different proteases for enzymolysis, can also help to obtain robust results.

Studies have shown that casein contains a varieties of amino acid sequences with immunocompetent activities in their inactive form [6]. After hydrolysis with a suitable protease, the related immunologic active fragments can be released, and it could be used as a drug or an ingredient in research and food production [7]. Anne isolated and purified the enzymatic hydrolysate from casein and found that the smaller molecular weight components inhibited the secretion of the pro-inflammatory factor Tumor necrosis factor-α (TNF-α) by lymphocytes, thereby weakening the allergic and inflammatory responses [8]. Another study also found that proline-rich polypeptides isolated from sheep colon whey have regulatory properties that stimulate or inhibit immune responses [9]. Furthermore, a nine-peptide fragment, VESYVPLFP, isolated from the chymotrypsin digests of sheep milk, has shown immunoregulatory activity in mice similar to naturally occurring proline-rich polypeptides [10]. According to the reports, the peptide VEPIPY obtained from casein enzymatic hydrolysis can promote immune stimulation [11]. For instance, Parma and collaborators collected the sterile and the bacterial mouse bone marrow pre-macrophages, and studied *in vitro* the effect of β-CN(193-209) on the cellular function of macrophages. The results showed that this peptide could enhance the expression of compatibility complex II antigen, the main component in macrophages, stimulating cytokines release and improving the phagocytosis of macrophages [12]. Moreover, short peptides with a molecular weight of less than 3 kDa are more potent to possess biological activities [13]. However, it remains unclear if the peptide produced by digestion and decomposition in the gastrointestinal tract has immune-active activities and if it can be transported and absorbed by the intestinal tract.

Food functional factors can exert their biological activity only if being absorbed and reaching the target organs. The critical point of this process is to resist the hydrolysis of enzymes in the gastrointestinal tract and be absorbed entirely by small intestinal epithelial cells [14]. However, it remains unexplored whether bioactive peptides can completely pass through the cell membrane and how they pass through small intestinal epithelial cells. The human colorectal adenocarcinoma cell (Caco-2) cell line is derived from human colorectal cancer and has many similar morphological and functional characteristics compared to mature intestinal cells. Furthermore, Caco-2 cells had to brush border epithelial enzymes on the apical side (AP) surface, and a similar expression of transport system vectors and metabolic enzymes was found in normal intestinal epithelial cells. Because of the close correlation between peptide absorption in the human intestine and the Caco-2 cell monolayer cell model, this method has become a standard screening tool for predicting drug absorption and transport mechanisms in the human intestine [15]. Therefore, the presence and absorption of the substance *in vivo* could be investigated on the Caco-2 cell model [16]. Some studies have revealed that intestinal transportation mechanisms are different for the individual peptides, depending on some factors, including the length of the peptide, the hydrophobic properties, and the charge characteristic of the peptides [17].

Here, bovine casein was digested by the simulating human gastrointestinal environment to detect immune activity and identify the immune-active peptides through LC-MS/MS. In addition, the absorption forms and mechanisms of immune-active peptides were investigated using the Caco-2 cell model.

## 2. Materials and Methods

### 2.1. Chemicals 

B-lymphocyte (Baili Biotechnology Co., Ltd., ShangHai, China.), TNF-αEnzyme-linked immunosorbent assay (ELISA) kit (BIO-SWAMP, WuHan, China); Arg-Tyr-Pro-Leu-Gly-Tyr-Leu (RYPLGYL), Lys-His-Pro-Ile-Lys-His (KHPIKH), Lys-His-Pro-Ile- Lys (KHPIK), Phe-Phe-Ser-Asp-Lys (FFSDK), Tyr-Gly-Gly (YGG, Hangzhou Dangang Peptide Synthesis Biology Co., Ltd..HangZhou, China) Human colorectal adenocarcinoma Caco-2 cell line were purchased from the Wuhan species collection center (Wuhan, China). DMEM high glucose medium, fetal bovine serum, penicillin-streptomycin (10,000 U/mL), non-essential amino acids, and trypsin-0.02% EDTA digestion solution were obtained from Hyclone (GE Healthcare, Logan, UK). Gly-Pro was provided from Shanghai Top Technology Co., Ltd. (Shanghai, China). phenylarsine, sodium deoxycholate, MK-571 sodium salt, sodium azide, verapamil, fluorescent yellow, dimethyl sulfoxide (DMSO), and 3,4,5-dimethylthiazol- 2,5-diphenyl-tetrazolium bromide (MTT) were purchased from the Sigma Chemical Co. (Madrid, Spain). In total, 12-well Transwell plates and other types of cell culture plates were purchased from Corning Incorporated. All other reagents were of analytical grade.

### 2.2. Simulated Gastrointestinal Digestion 

The simulated gastrointestinal digestion was performed according to the method described by Linda and collaborators with some modifications [18]. Briefly, the casein solution was adjusted to pH 2.0 with hydrochloric acid. Then, the mixture oscillated in a water bath at 37 °C for 10 min. Next, the artificial gastric juice was added, following the ratio of enzyme/substrate (E/S) being equal to 1:50 and placed in a water bath oscillator at 37 °C for digestion. Afterwards, samples were taken every 15 min within 0–1 h and every 1 h within 1–5 h. In addition, the retained samples were digested for 2 h, oscillated in a 37 °C water bath oscillator for 10 min, and then the artificial intestinal liquid was added. The further experimental steps were the same as described above. After the samples were heated at 100 °C for 10 min to inactivate the enzymes, the pH was adjusted to 4.6, and the casein precipitate was removed by centrifugation at 5000× *g*/min at 4 °C for 20 min. Finally, the pH of the upper layer solution was adjusted to 7.0 and stored at −20 °C for subsequent analysis. The simulated gastric and intestinal fluids were prepared according to the Chinese Pharmacopoeia and were prepared and maintained at 37 °C during experiments. Furthermore, the digestion samples were divided into two parts by ultrafiltration with a Molecular Weight Cut Off (MWCO) of 5 kDa membrane.

### 2.3. Immune Activity Assay

The B-lymphocytes were purchased and placed in a carbon dioxide incubator at 37 °C, 5% CO_2_, and 90% humidity for adaptive culture for 3–4 h. After the excess medium was removed, fresh medium was added.

#### 2.3.1. MTT Assay to Evaluate B Lymphocyte Proliferation

The Thiazolyl Blue Tetrazolium Bromide (MTT) method was used to measure cell growth inhibition. For that, B lymphocytes of mice in the logarithmic growth stage were collected and added to a 96-well plate with the cell density adjusted to 5 × 10^6^ cells/mL. Normal saline was used as a control group, and the experimental groups were digested for samples less than 5 kDa and larger than 5 kDa after treatment. In this experiment, 3 parallel wells were used for each group and cultured in a 5% CO_2_ cell incubator at 37 °C for 48 h. The MTT solution was added to each well for 4 h before the end of the experiment and centrifuged for 5 min at a speed of 1000 r/min in a flat centrifuge 4 h later. The supernatant was discarded, and an appropriate amount of dimethyl sulfoxide was added to each well. After complete color development, the absorbance of each well was measured at 570 nm using a Bio-Rad 550 enzyme-linked immunosorbent assay. The B lymphocyte proliferation rate was calculated according to the following formula:

(1) $$Cell\;proliferation\;rate(\%)=\left(A_{e}-A_{c}\right)/A_{e}\times 100\%$$

where: *A*—The detected OD value; *A_e_*—OD value of the experimental group; *A_c_*—OD value of the control group.

#### 2.3.2. Detection of TNF-α

In total, 100 μL of B-lymphocytes with a cell density of 1 × 10^5^ seeded into a 96-well plate and 100 μL of RMPI-1640 were added, and then 50 μL of peptide fragments with the concentrations from 0 mM to 10 mM were added. Three parallel wells were set in each group. After culturing for 48 h under the condition of 37 °C and 5% CO_2_, experiments were conducted according to the operating procedures of the instructions of the TNF-α enzyme-linked immunosorbent assay kit [10].

### 2.4. RP-HPLC Detection of Immunologically Active Peptides

Reverse phase high-performance liquid chromatography (RP-HPLC) was used to analyze the composition of peptides passed through the Caco-2 cell membrane. The detection conditions used in this experiment were listed in Table 1. The elution parameters were as follows: the mobile phase B was ultra-pure water containing 0.1% Trifluoroacetic acid (TFA), and mobile phase A contained acetonitrile supplemented with 0.1% TFA. Adopting a gradient elution method, in detail, at 0.01 min, mobile phase A was 5%; at 25 min, mobile phase A was 70%. The absorbance of RP-HPLC was detected at 220 nm. The peak yields of the five electroactive peptides at the concentrations of 0.5 mM, 0.2 mM, 0.1 mM, 0.05 mM, and 0.025 mM were detected. The concentration was set as the abscissa and the peak area was set as the ordinate to draw the standard curves of the different peptide segments.

### 2.5. LC-MS/MS Detection of Immunologically Active Peptides

Mass spectrometry technology was mainly applied to the digestive peptidomics of protein. The mass spectrometric detection system is Q Exactive, equipped with nanoESI in the positive ion mode, for the search and identification of the characteristic peptide trypsin hydrolysate. The ionization source conditions were set as follows: capillary voltage, 3.5 kilovolts; source temperature, 275 °C; the collision gas is high purity nitrogen. The quantitation mode was performed in DDA mode. The other parameters are shown in Table 2. The MS raw file was processed and converted by the MM File Conversion software to an MGF format file, which was then used by MAS-COT (http://www.matrixscience.com/) to retrieve the uniprot database. Two-stage mass spectrometry was also performed for the identification of three peptide fragments (YPFPGPI, FFSDK, and KHPIK).

### 2.6. Transportation Assay

Caco-2 cells were maintained in an incubator at 37 °C, with 5% CO_2_ and relative humidity of 90% until the number of cells accounted for 80–90% of confluency [19]. After that, the medium was added to obtain a cellular suspension with 4 × 10^5^ cells/mL, which was seeded on a 12-well Transwell plate added to the AP side of the 0.5 mL cell suspension, and the Basal lateral (BL) side by adding 1.5 mL of cell culture medium after one day after the exchange, Then, the fluid was changed every other day for 6 days, and the fluid was changed every day until the culture was completed. Cell membrane integrity and other indicators must be tested to successfully establish Caco-2 cell models. The main detection indicators were: (1) cell morphology observation; (2) the cell membrane compactness using fluorescent yellow test; (3) the detection of single-cell membrane resistance [20]; (4) alkaline phosphatase activities at the top and the basal side of the cell membrane were detected respectively to preliminarily determine the polarity of it.

The *Papp* formula of fluorescence is as follows:

(2) Papp=dQdtA×C0

where: *C_0_*—Initial concentration of fluorescent yellow (mg/mL); *A*—Membrane surface area (cm^2^); *dQ*/*dt*—Transport of fluorescent yellow per unit time; *Papp*—Apparent permeability coefficient.

The transport experiment was conducted with KHPIK to optimize the main conditions including concentration, contact time, pH, and transport direction, which could affect the transmembrane transport of bioactive peptides. 

### 2.7. Transport Mechanism of the Immunologically Active Peptide

#### 2.7.1. Study on the Absorption Mechanism of Peptides

After 0.5 mL of 10 mM Gly-Pro, 0.1 mM sodium deoxycholate and 0.025 mM phenylarsine oxide were added to different wells on the AP side, and the Transwell plates were incubated in a CO_2_ incubator for 30 min and followed by the removal of these compounds. The peptides were then added to the AP side, and Hank’s Balanced Salt Solution (HBSS) buffer was added to the BL side, and samples were collected after 2 h.

#### 2.7.2. Study on Peptide Efflux

In total, 0.5 mL of 0.05 mM MK-571 sodium salt, 0.1 mM verapamil, and 10 mM sodium azide were added to the AP side hole of the Transwell plate and incubated in a CO_2_ incubator for 30 min. After this incubation, the compounds were removed, the peptide was added to the AP side, and HBSS buffer was added to the BL side for 2 h.

### 2.8. Statistical Analysis

The amino acid sequence was analyzed by Liquid chromatography-mass spectrometry/mass spectrometry (LC-MS/MS) sequential software and PEAKS 7.0 software. The data obtained was visualized using GraphPad Prism 6.01 software and analyzed using SPSS 22.0 software for significance analysis (*p* < 0.05) via one-way analysis of variance (ANOVA).

## 3. Results

### 3.1. Identification of Immune-Active Peptides in the Gastrointestinal Hydrolysate In Vitro

**Figure 1:**
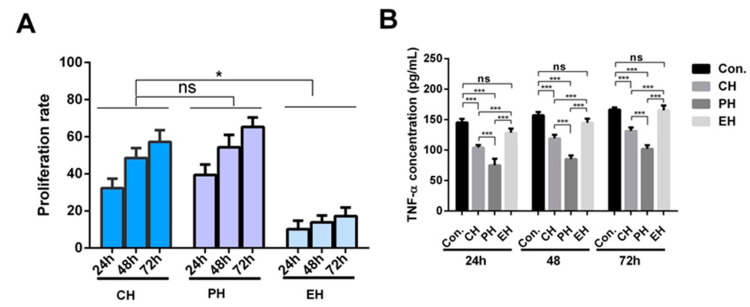
Effect of casein hydrolysate on B lymphocytes stimulation. (**A**) B lymphocyte proliferation activities of peptides after 24 h. (**B**) Effect of casein hydrolysis on secretions of TNF-α in B lymphocyte. In the figure: CH-Casein hydrolysate, PH-Permeate hydrolysate, EH-Entrapment hydrolysate. Data are presented as the mean ± SD (*n* = 6). ns, *p* > 0.05; *, *p* < 0.05; ***, *p* < 0.001 versus control group.

The immunologic activity of the hydrolysate was explored. The results demonstrated that the casein hydrolysate and the permeate hydrolysate both promoted the proliferation of B-lymphocytes, indicating that the active immunologic mixture in casein hydrolysis was mainly less than 5 kDa (Figure 1). The majority of immunologically active peptides are hydrophobic peptides according to the previous studies. The peptides with less than 5 kDa in the ultrafiltration filtrate were analyzed by Liquid Chromatography- Quadrupole/Time of Flight Tandem Mass Spectrometer (LC-Q-TOF MS/MS) mass spectrometry. Furthermore, the identified peptides were mapping to the records in MBPDB database (http://mbpdb.nws.oregonstate.edu). Table 3 shows the peptides with immunoregulatory activity. We found that the active peptide segments, KHPIKH and RYPLGYL, are derived from α_S1_-casein, and FFSD is derived from κ-casein. Furthermore, KHPIK and KHPIKH differed only in one amino acid, and KHPIK was also found to be active by highly credible peptide fragmentation pattern and matching in LC-MS/MS sequence software mascot 2.3.0. These hydrolysis-resistant peptides may exert immunoregulatory activity *in vivo*. Since polypeptides containing less than three amino acids cannot be detected by mass spectrometry, these kinds of peptides could not be identified in the casein-derived hydrolysate. Herein, four identified peptides including KHPIK, KHPIKH, RYPLGYL, FFSDK, and a triplet peptide YGG derived from κ-Casein with a strong proliferation effect on peripheral blood lymphocytes [18] were synthesized to determine the transport characteristics and mechanism in a Caco-2 cell monolayer.

### 3.2. Immunoactivity Test of the Five Synthetic Peptides

**Figure 2:**
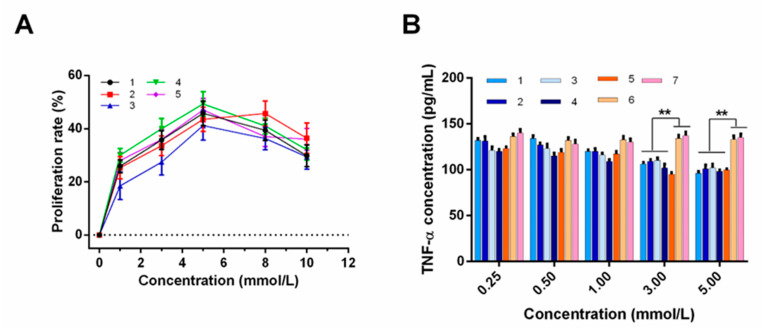
Immunoactivity test of the five synthetic peptides. (**A**) Cell proliferation rate at different concentrations of peptides. (**B**) Effect of different peptide concentrations on the secretion of TNF-α in B lymphocytes. Data are presented as the mean ± SD (*n* = 6). **, *p* < 0.01 versus control group.1-RYPLGYL, 2-KHPIKH, 3-KHPIK, 4-FFSDK, 5-YGG, 6-control group, 7-blank group.

The measurement of the effect of RYLGY on cell proliferation showed no significant difference with the control group, which indicated that the peptide did not promote the proliferation of B-lymphocytes. Therefore, RYLGY was selected as a control group for the immune activity experiment. The B-lymphocyte proliferation rates of the five synthesized short peptides were detected, and the results are shown in Figure 2A. When the concentration of the five peptide fragments was between 0–5 mM, the B lymphocyte proliferation rate changed depending on the peptide concentration (*p* < 0.05). As the concentration was greater than 5 mM, the proliferation rate decreased because high concentrations might induce cell damage and peptide inhibition. The immune-active peptides detected in our study had a similar proliferation rate with the immune-active peptide GYPMYPLPR used in a previous study [23]. Therefore, a concentration of 5 mM was kept for the stimulation of TNF -α secretion test of B-lymphocytes.

The synthesized five short peptides were treated with cell lines to detect the TNF-α secretion, and the results are shown in Figure 2B. When the peptide concentration was 3 mM and 5 mM, significant differences were observed comparing with the blank and control groups. Furthermore, the higher peptide concentration, the lower level of TNF-α (*p* < 0.05). Studies showed that other immunological active peptide fragments had similar ef-fects. For instance, the hazelnut hydrolysate containing the immunologically active peptide PEDEFR significantly inhibited the secretion of TNF-α [10]. 

### 3.3. Construction of the Caco-2 Cell Model

**Figure 3:**
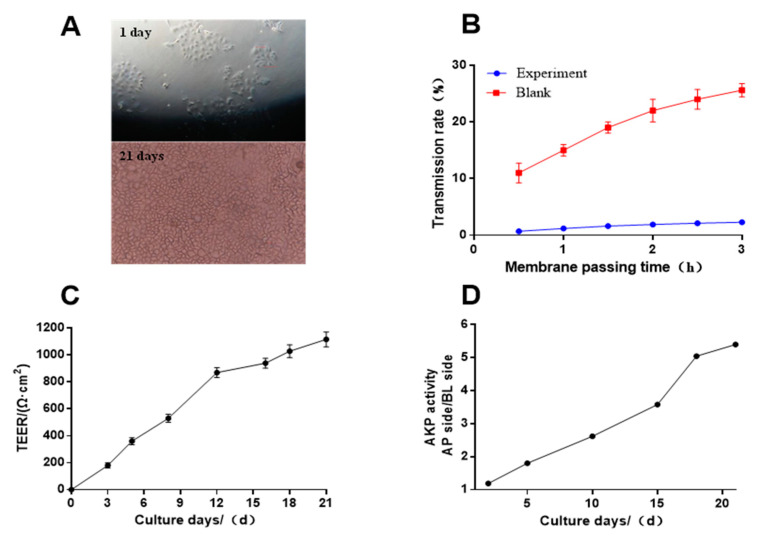
Evaluation of the monolayer density. (**A**) Caco-2 cells growth (10×). (**B**) Effect of transportation time on transmittance. (**C**) Effect of culture time on cell membrane resistance. (**D**) Effect of culture time on Alkaline phosphatase (AKP) activity ratio.

The Caco-2 cell monolayer was evaluated at four aspects. The Caco-2 cells grew adherent to the wall after 21 days of culture and observed using an optical microscope (Figure 3A). The cells grew evenly and were closely connected.

Figure 3B shows the density of the monolayer film determined by fluorescence yellow transmittance. The transmittance of fluorescence yellow in the experimental group was increased by only 1.6% from 0.5 h to 2.5 h, which was significantly lower than 24.10% in the control group. Moreover, the *Papp* of fluorescence yellow was 6.07 × 10^−7^ cm/s, suggesting that the Caco-2 cell membrane was already very dense and could be used to understand the transport mechanism [24]. The transepithelial electrical resistance (TEER) values are shown in Figure 3C. After 12 days, the value of TEER was greater than 700 Ω cm^2^, proving that the cell lines model met the experimental conditions. The results in Figure 3D showed that the ratio of alkaline phosphatase activity between AP and BL sides on the 21st day was 5.45 times higher than after 5 days. Therefore, it was confirmed that the Caco-2 monolayer had formed a polarization, which can be used to study the cellular absorption mechanism of substances.

### 3.4. The Optimal Transport Condition of the KHPIK Peptide

**Figure 4:**
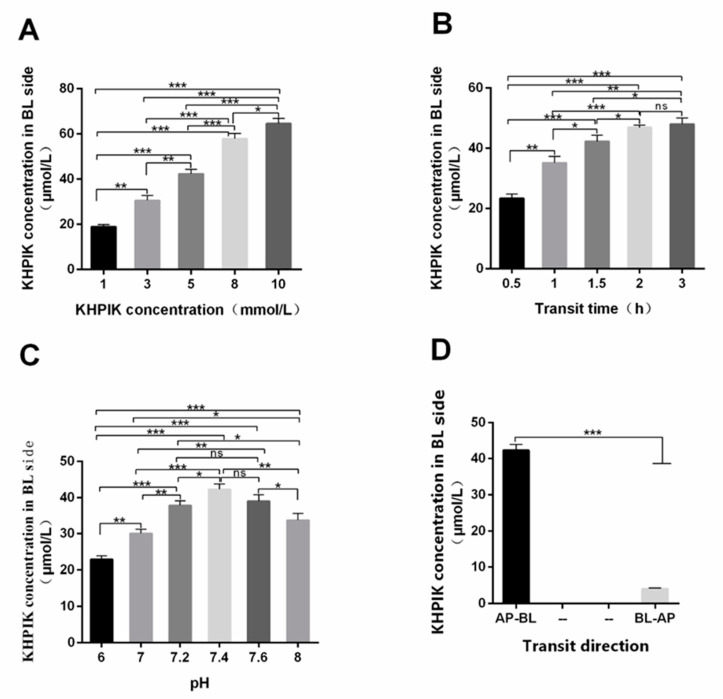
The optimal transportation of conditions of KHPIK. (**A**) effect of KHPIK concentration on transportation. (**B**) effect of incubation time on transportation. (**C**) effect of KHPIK pH on transportation. (**D**) effect of KHPIK transit direction on transportation. Data is presented as the mean ± SD (*n* = 6). ns, *p* > 0.05; *, *p* < 0.05; **, *p* < 0.01; ***, *p* < 0.001 versus the control group.

The results in Figure 4A demonstrated that the KHPIK concentration on the BL side of the Caco-2 monolayer cell membrane increased with concentration in the AP side, indicating that the transport amount of polypeptide on the Caco-2 cell model was concentration-dependent within the range of 1–10 mM. The experimental concentration, 5 mM, was used in this study to avoid the saturation of the transport quantity. It can be observed in Figure 4B that when the time was between 0.5 and 2 h, the amount of KHPIK transported increased rapidly, whereas when the time was between 2 h and 3 h, the amount of KHPIK across the membrane increased slowly. This can be explained because the concentration differences in between the two cell monolayer sides decreased with the increase in transport amount, leading to the slow transport rate. At the same time, the cell activity decreased with contact time without changing the culture medium. Taking into account these results, the transportation time was defined as 2 h. Furthermore, the results also demonstrated that when pH was 6.0, the transmembrane transport capacity of KHPIK on the BL side was 23.14 μM (Figure 4C). On the other hand, the transport capacity was greater than 30.01 μM when pH was 7.0–8.0 and reached 44.35 μM when pH was 7.4, which was significantly different from previous studies (*p* < 0.05). Given the pH of human small intestinal cells is around 7.4, this can affect cellular physiological activity. The results also revealed that the transport quantity of KHPIK from the BL-AP side was significantly lower than that from the AP-BL side (Figure 4D) (*p* < 0.05), indicating that KHPIK is easier to be transported and absorbed from the top of intestinal cells to the basal side, so the transport direction was from AP to BL (AP-BL) side. However, a small amount of peptides transport from the BL-AP side suggests that the polypeptide transport may be related to efflux.

### 3.5. RP-HPLC Analyzing of the Immune-Active Peptides after Transportation

RP-HPLC was used to quantify and qualify the peptides in samples obtained from the BL side after transportation. The permeability coefficient was calculated and the hydrolysis of the peptide segment was preliminarily determined The red line in the drawing corresponds to the liquid chromatograms of five standard bioactive peptides with a concentration of 0.5 mM. 

**Figure 5:**
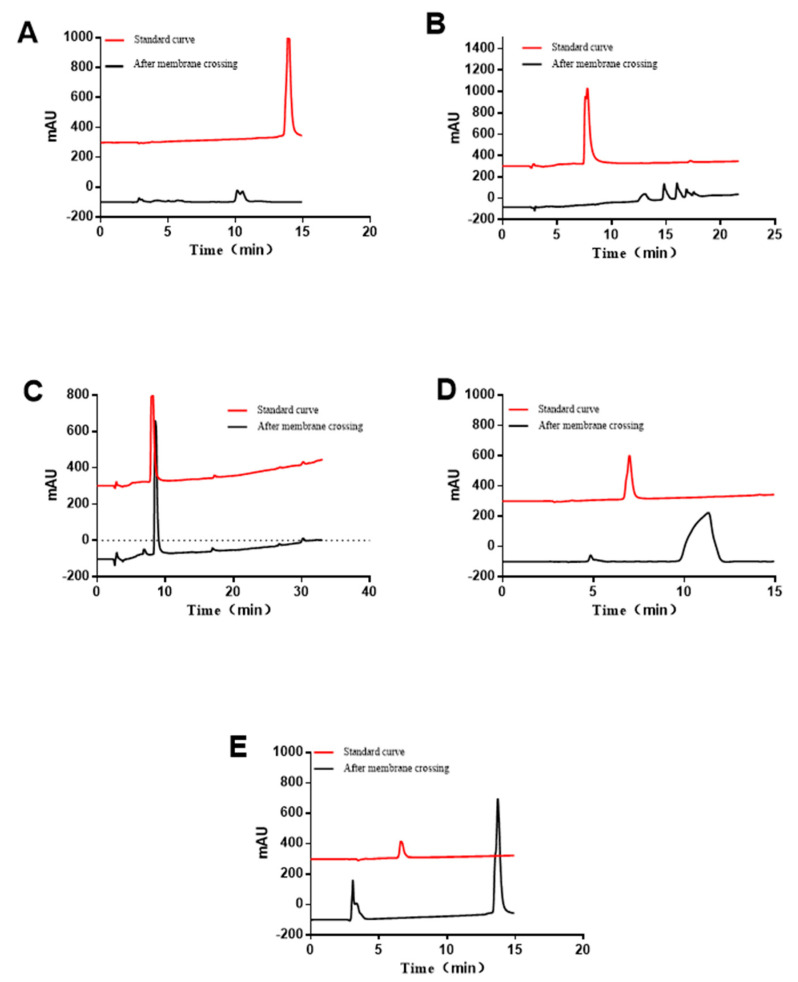
The composition of the five peptides analyzed by RP−HPLC after passing through the Caco−2 cell monolayer. (**A**) RYPLGYL (**B**) KHPIKH (**C**) KHPIK (**D**) FFSDK (**E**)YGG.

According to the corresponding standard curves, the *Papp* values of RYPLGYL, KHPIKH, KHPIK, FFSDK, and YGG on Caco-2 cells were 1.61 × 10^−7^ cm/s, 2.60×10^−7^ cm/s, 3.22 × 10^−7^ cm/s, 4.91 × 10^−7^ cm/s, and 5.12% × 10^−7^ cm/s. The results suggest that the shorter the peptide segment, the easier it is to penetrate the Caco-2 cell membrane. After passing through the Caco-2 cell membrane, the five immunologically active peptides were decomposed to greater or less extent and thereby showed other peaks besides their own (Figure 5). The results indicated that the polypeptide may be hydrolyzed by a brush edge enzyme system on the surface of the Caco-2 cell membrane to produce a new fragment, reducing the transport of the parent peptide on intestinal epithelial cells.

### 3.6. Study on the Absorption Mechanism of the Immune-Active Peptides

**Figure 6:**
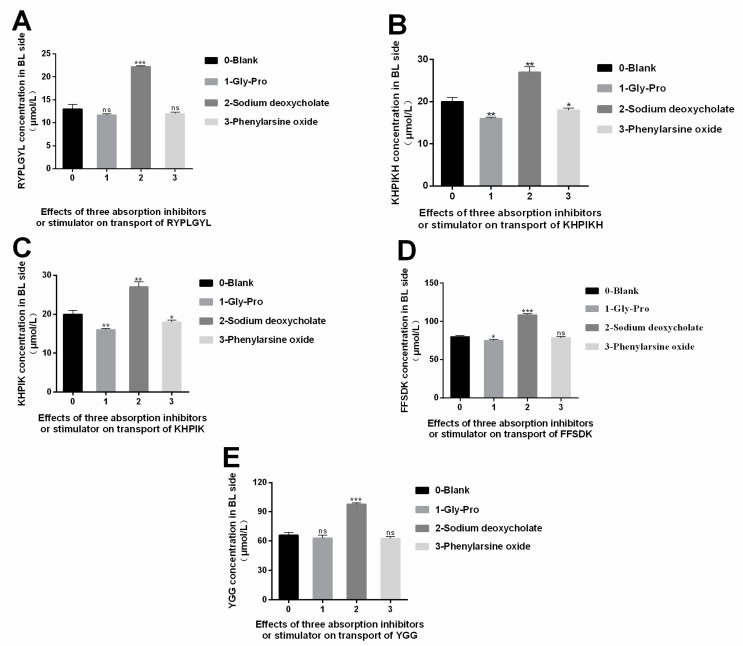
Effects of three absorption inhibitors or stimulator on peptide segment transport. (**A**) RYPLGYL. (**B**) KHPIKH. (**C**) KHPIK. (**D**) FFSDK. (**E**) YGG. Data are presented as the mean ± SD (*n* = 6). *, *p* < 0.05; **, *p* < 0.01; ***, *p* < 0.001 versus control group.

It has been described that polypeptides pass through cell membranes in the following ways: intercellular transport, passive transport, active carrier transport, endocytosis, and efflux [25]. In passive transport, substances pass through cell membranes based on the principle of similar miscibility, and membrane proteins are not involved in the transport. In carrier transport, a carrier protein promotes the transfer of the substances on the cell membrane. During endocytosis, the substances enter the cells as vesicles without binding to the cell membranes. In carrier transport, a carrier protein promotes the transfer of the substances on the cell membrane. On the other hand, bypass transport is the transport of substances into cells through tight junctions between cells. The inhibitors or stimulators are usually used to investigate the transport mechanisms. As a substrate Peptide transporter 1 (Pep T1), Gly-Pro can competitively bind to Pep T1 with the other target peptides [26]. Therefore, Gly-Pro is often used as an inhibitor to study the role of transport carrier. Phenylarsine oxide is an inhibitor that inhibits the endocytosis of substances in the cross-cell process. Sodium deoxycholate is a bypass transport promoter that can change the degree of tight junction between cells, thus strengthening the bypass transport permeability. Therefore, the Gly-Pro, phenylephrine oxide, and sodium deoxycholate, were added to determine the main absorption pathway of the five immunologically active peptides in the intestinal tract (shown in Figure 6). We observed that Gly-Pro could inhibit the transport of RYPLGYL, KHPIKH, KHPIK, FFSDK, and YGG on the BL side after 2 h, but no significant differences were detected (*p* > 0.05), indicating that the carrier transport of peptides may not be the main transport mechanism for these peptides (Figure 6). Phenylarsine oxide is an inhibitor compound that inhibits the endocytosis of substances. As shown in the Figure 6, compared to the blank group, phenylarsine oxide, had a small effect on the transmembrane transport of these peptides, and no significant differences were detected (*p* > 0.05). Therefore, the results indicate that endocytosis is not the main transport mechanism. However, when sodium deoxycholate was added, a significant increase in the transmembrane transport of these peptides was observed (*p* < 0.05). Overall, the results suggest that bypass diffusion is the main mechanism used to transport RYPLGYL, KHPIKH, KHPIK, FFSDK, and YGG in the intestinal epithelial cells.

### 3.7. The Efflux Mechanism of the Immune-Active Peptides

Multidrug resistance protein (MRP) and P-glycoprotein in Caco-2 cells are energy-dependent efflux transporters that use Adenosine triphosphate (ATP) as an energy source. P-glycoprotein on the AP side of the Caco-2 cells and the MRP2 in MRP can efflux cellular endogenous and exogenous substances to the AP side, resulting in less absorption. It has been stated that MK-571 sodium salt inhibits the efflux of MRP2, and on the other hand, verapamil can inhibit the efflux of P- glycoprotein. Furthermore, some studies have described that sodium azide is an inhibitor that can inhibit ATP production on the cell membrane. The inhibitors described above were used in this study to understand the underlying efflux mechanism of the immune-active peptides [27]. 

**Figure 7:**
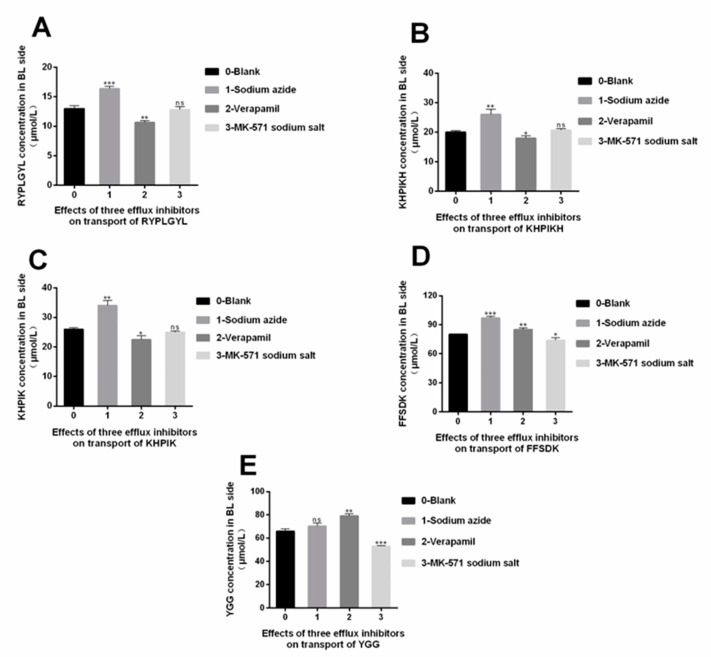
Effects of three efflux inhibitors on peptide segment transport. (**A**) RYPLGYL. (**B**) KHPIKH. (**C**) KHPIK. (**D**) FFSDK. (**E**) YGG. Data are presented as the mean ± SD (*n* = 6). ns, *p* > 0.05; *, *p* < 0.05; **, *p* < 0.01; ***, *p* < 0.001 versus control group.

The results obtained in Figure 7 showed that after adding MK-571 sodium salt, and verapamil, no significant differences in transmembrane transport of RYPLGYL, KHPIKH, and KHPIK were detected (*p* > 0.05), indicating that MRP2 and p glycoprotein did not interfere with the efflux of these three peptides. On the other hand, the concentration of RYPLGYL, KHPIKH, and KHPIK on the BL side increased significantly after adding sodium azide (*p* < 0.05), indicating that the transport of these three peptides depends on the consumption of ATP. Furthermore, the results also demonstrated that adding MK-571 sodium salt had no significant or differenct effects on the transport quantity of FFSDK compared with the blank group (*p* > 0.05). However, the transmembrane transport quantity of FFSDK significantly increased after adding verapamil and sodium azide (*p* < 0.05), indicating that FFSDK can use p-glycoprotein as a carrier and consume ATP to promote this transport. Moreover, Figure 7 showed that verapamil could significantly promote the transport of YGG from the AP side to the BL side (*p* < 0.05), indicating that the YGG efflux during absorption is mainly mediated by P- glycoprotein. However, no significant differences were observed in the transport amount after adding MK-571 (*p* > 0.05). Additionally, the concentration of YGG in the BL side after adding sodium azide was slightly higher than in the blank group, suggesting that some ATP production was inhibited, impacting the transport amount.

### 3.8. Integrity Analysis of the Peptide Fragment Passing through the Membrane

**Figure 8:**
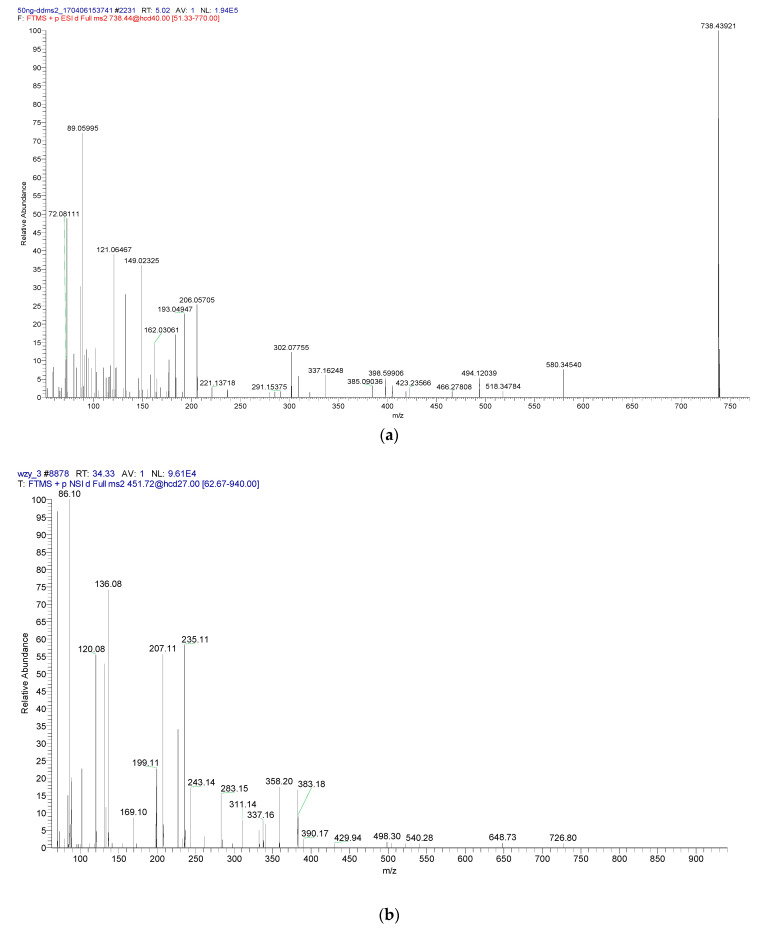

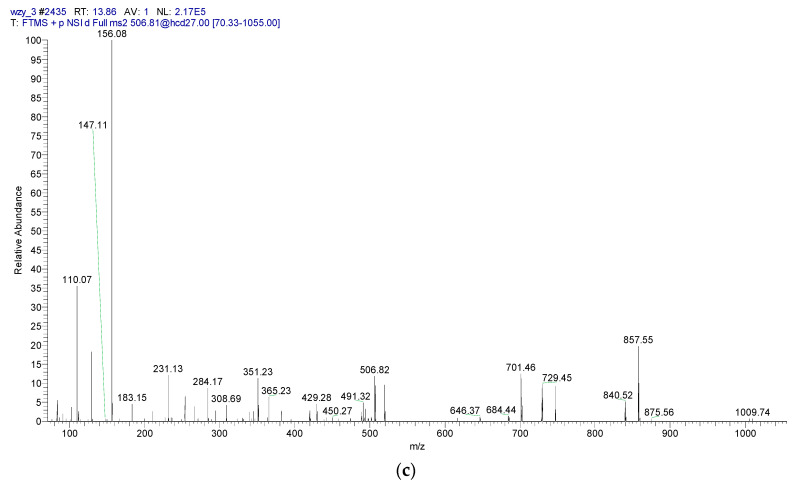
RYPLGYL, KHPIK, and FFSDK LC-MS result analysis. (**a**) LC/MS of RYPLGYL passing through membrane liquid. (**b**) LC/MS of KHPIK passing through membrane liquid. (**c**) LC/MS of FFSDK passing through membrane liquid.

According to the RP-HPLC spectrum, there is degradation of peptides after passing through the cell membrane (Figure 5). Further, the degradation degree of the peptide was determined by LC/MS (Figure 8). Using the SPEAKS software to obtain a very reliable peptide fragment cleavage mode and combining these results with the original peptide fragment connection mode, the peptide fragments are inferred and shown in Table 4. The results demonstrated that RYPLGYL is resistant to the enzymatic hydrolysis of intestinal epithelial cells and could be transported through the cell membrane with a complete form to exert the immune function. However, only a small fragments of intact KHPIK and FFSDK passes through Caco-2 monolayer cells. Most of the KHPIK and FFSDK were hydrolyzed into KHP and FFS by the brush border enzyme, respectively. In addition, the peptide fragment SDK, derived from FFSDK, belongs to a κ-CN terminal and this terminal is difficult to be hydrolyzed. Although different immune-active peptides may be hydrolyzed in the transportation stage to different extents, some amounts of intact immune-active peptides could still be transfered from the intestinal epithelial and may exert immunological activities in the local immunity system.

## 4. Discussion

*In vivo* evaluation of bioactive peptides release and absorption in human beings are difficult to perform for the generation of great endogenous peptides. Hence, several *in vitro* digestion methods with simulating gastrointestinal tract were developed. Here, we identified 37 potential immune-active peptides by the simulated gastrointestinal digestion. Given the complexity of digestion and absorption process, it is difficult to trace the food-derived peptides in the gastrointestinal tract. Most studies developed the Caco-2 monolayer membrane to investigate the adsorption mechanisms.

Relevant reports have shown that the polypeptides with amino acids in the range of 2–9 peptides containing more than 3 amino acids are more easily hydrolyzed, resulting in a low transport amount of the polypeptide by the cell membrane [28]. The main reason is because of the hydrolysis of the enzyme system on the cell membrane surface; the terminal protection exists in the transport of the polypeptide, so the integrity of the Caco-2 cell membrane is not damaged during the transport process [18]. The peptide segment containing proline residue had stronger resistance to the enzymatic hydrolysis of the brush border enzyme system, but the segment containing leucine at the nitrogen end was more likely to be hydrolyzed [29]. The validation results showed that the Caco-2 cell model had been successfully constructed, and the transport and absorption mechanisms of peptides could be studied in the Caco-2 cell model. Taking KHPIK as an example, the transport conditions of peptide liquid on cells were determined: transport concentration of 5 × 10 ^− 3^ M, pH 7.4, transport in a direction from AP to BL, and transport time of 2 h. The absorption mechanisms of the five bioactive peptides in the Caco-2 cell model were all in the form of bypass transport. However, the transportation of RYPLGYL, KHPIK, and KHPIKH might be companied with efflux, which may require ATP to provide energy. The efflux of YGG during absorption was mainly mediated by P- glycoprotein. On the other hand, the FFSDK efflux was a transport process mediated by P- glycoprotein, and ATP was used as an energy source. Additionally, the LC-MS/MS results showed that some peptides (RYPLGYL, FFSDK, and KHPIKH) passed through the Caco-2 monolayer cell membrane and maintained their structural form. However, some were hydrolyzed into small-molecule polypeptides by the brush border enzyme system on the cell membrane. Rishika and collaborators studied the transport modes of three bioactive peptides derived from milk protein. They found that the transmembrane transport ratio of VLPVPQK was higher than TPFPG and RPGFSPFR since the heptapeptide was transported into the body as a carrier, and the primary mode of intestinal absorption of pentapeptide and nonapeptide was bypass transport [30]. Additionally, Pappen and collaborators demonstrated that a polypeptide comprising eight amino acids is likely transported by bypass through intestinal epithelial cells [31]. Furthermore, Adson and colleagues found that DFG mainly passed through intestinal epithelial cells in the form of bypass transport [32]. Overall, these previous studies have demonstrated that the absorption mechanism of bioactive peptides in the Caco-2 cell model is mostly through bypass transport, and all of them can pass through the Caco-2 monolayer cell model in an intact form. 

Although the immunologically active peptides can completely cross the Caco-2 cell membrane, its interaction with related peptidases (such as DPP-IV) and transporters (P-glycoprotein) during transport remains to be further verified. In fact, the effects of other food substrates including starch, polysaccharide, lipid etc. on digestion and absorption should also be investigated for real food ingestion.

## 5. Conclusions

Casein hydrolysis presented the immune activity through the simulated gastrointestinal method, and these results were identified by the LC-MS/MS. The five peptides in the study, RYPLGYL, KHPIKH, KHPIK, FFSDK, and YGG, were synthesized, and the results proved that they had immunological activity by promoting the proliferation rate of B lymphocytes and reducing the secretion of TNF-α. Furthermore, the transport mechanism of these five peptides occurred through bypass on the Caco-2 monolayer cell model. On the other hand, the Papp values of RYPLGYL, KHPIKH, KHPIK, FFSDK, and YGG within 2 h were 1.61 × 10^−7^ cm/s, 2.60 × 10^−7^ cm/s, 3.22 × 10^−7^ cm/s, 4.91 × 10^−7^ cm/s, and 5.12% × 10^−7^ cm/s. The LC-MS/MS identified the permeant fluid and found that one part of this peptide was completely absorbed by intestinal epithelial cells, and the other was hydrolyzed. These suggest that these immunologically active polypeptides could be absorbed and may play a role in addition to its enteric cavity. It is expected that this conclusion can be improved through animal experiments.

## Figures and Tables

**Table 1:** Chromatographic conditions.

Chromatographic Column C18.	Wavelength	Sample Size	Flow Rate	Mobile Phase A	Mobile Phase B
4.6 mm 5 μm	220 nm	20 μm	1.0 mL/min	0.1%TFA acetonitrile	0.1% TFA ultrapure water

**Table 2:** Mass Spectrometer Parameters.

Primary Mass Spectrometry Parameters	Resolution	AGC Target	Maximum IT	Scan Range	
	7000	3e6	40 ms	50 To 750 *m*/*z* (YPFPGPI) 50 to 1800 *m*/*z* (FFSDK, KHPIK)	
Two-stage mass spectrum parameters	Resolution	AGC target	Maximum IT	TopN	NCE/Stepped
	17500	1e5	60 ms	20	27

**Table 3:** Immunomodulatory peptides identified in simulated GI digestion.

Sequence Identified	Source	Activity
ALARPKHPIKHQ	α_S1_ 18–23	KHPIKH [21]
VAVALARPKHPIKHQGLPQEVLNENLL		
RYPLGYLEQLLR	α_S1_ 105–110	RYPLGYL
KDDVPSERYLGYLEQLLR		
RYLGYLEQLLR		
KHIQKEDVPSERYLGYLEQLLR		
PNSVEQKHIQKEDVPSERYLGYLEQL		
LAYFYPEL	α_S1_ 157–164	LAYFYPEL
EPMIGVNQELAYFYPEL		
EPMIGVNQELAYFYPELF		
LAYFYPEL		
NPWDQ	α_S2_ 122–126	NPWDQ
PIVLNPWDQVK		
QGPIVLNPWDQVK		
QGPIVLNPWDQVKR		
YPFPGPI	β-casein 75–81 [8]	YPFPGPI
VYPFPGPIPN	β-casein 78–83	
LVYPFPGPIPN		
SLVYPFPGPIPN		
QSLVYPFPGPIPN		
VYPFPGPIPNSLPQ		
SLVYPFPGPIPNSLPQ		
AQTQSLVYPFPGPIPN		
AQTQSLVYPFPGPIPNSLPQ		
VYPFPGPIPNSLPQNIPPLTQT		
YQEPVLGPVR	β-casein 209–213	YQEPVLGPVR
YQEPVLGPVR		
LYQEPVLGPVR		
LLYQEPVLGPVR		
QEPVLGPVRGPFPIIV		
YQEPVLGPVRGPFPIIV		
LYQEPVLGPVRGPFPII		
YQEPVLGPVRGPFPIIV	β-casein 208–224 [22]	YQEPVLGPVRGPFPIIV
LYQEPVLGPVRGPFPIILYQEPVLGPVRGPFPIIV		
PGPIPN		
FFSDKIAK	κ-Casein 38–42	FFSDK

**Table 4:** KHPIK hydrolysis after mass spectrometry sequence.

Molecular Weight	−H_2_0	−NH_3_	Spectral Value	Seq	Credibility	Concentration (ppm)
391.54	370.75	374.29	337.18	YPL	*	6.33
434.54	412.52	417.84	395.09	RYP	*	4.79
448.66	430.76	431.95	423.2	PLGY	*	4.10
561.88	543.9	545.18	488.2	PLGYL	*	3.59
725.1	707.12	708.4	580.2	YPLGYL	*	9.21
881.32	863.34	864.62	738.4	RYPLGYL	*	10.74
380.54	362.56	363.83	358.20	KHP	*	16.62
356.54	338.56	338.83	311.14	PIK	*	12.47
493.76	475.78	477.05	390.17	KHPI	*	4.01
493.77	475.79	477.06	429.94	HPIK	*	3.36
621.98	604	605.27	648.73	KHPIK	*	2.59
399.54	381.56	382.84	365.23	FFS	*	8.62
348.44	330.48	331.73	308.69	SDK	*	5.47
495.66	477.68	478.95	450.27	FSDK	*	3.01
514.66	496.68	497.95	491.32	FFSD	*	5.36
642.88	624.90	626.17	646.37	FFSDK	*	2.59

* represents extremely credible.

## Data Availability

The data are available from the corresponding author.

## Abbreviations

LC-MS/MS, Liquid chromatography-mass spectrometry/mass spectrometry; HPLC, High-performance liquid chromatography; RYPLGYL, Arg-Tyr-Pro-Leu-Gly-Tyr-Leu; KHPIKH, Lys-His-Pro-Ile- Lys-His; KHPIK, Lys-His-Pro-Ile- Lys; FFSDK, Phe-Phe-Ser-Asp-Lys; YGG, Tyr-Gly-Gly; ACE, Angiotensin converting enzyme; TNF-α, Tumor necrosis factor-α; CN, Casein; VESYVPLFP, Val-Glu-Ser-Tyr-Val-Pro-Leu-Phe-Pro; VEPIPY, Val-Glu-Pro-Ile-Pro-Tyr; AP, Apical Side; ELISA, Enzyme-linked immunosorbent assay; DMEM, Dulbecco’s modified eagle medium; EDTA, Ethylene Diamine Tetra-acetic Acid; DMSO, dimethyl sulfoxide; MTT, Methyl thiazolyl tetrazolium; RPMI, Roswell Park Memorial Institute; OD, Optical Density; TFA, trifluoroacetic acid; HBSS, Hank’s Balanced Salt Solution; BL, Basal lateral; AKP, Alkaline phosphatase.

## References

[1] Duanis-Assaf D., Kenan E., Sionov R., Steinberg D., Shemesh M. (2020). Proteolytic Activity of Bacillus Subtilis upon κ-Casein Undermines Its “Caries-Safe” Effect. Microorganisms.

[2] Kim J.E., Lee H.G. (2021). Amino Acids Supplementation for the Milk and Milk Protein Production of Dairy Cows. Animals.

[3] Freund M.A., Chen B., Decker E.A. (2018). The Inhibition of Advanced Glycation End Products by Carnosine and Other Natural Dipeptides to Reduce Diabetic and Age-Related Complications. Compr. Rev. Food Sci. Food Saf..

[4] Xu Q., Hong H., Wu J., Yan X. (2019). Bioavailability of Bioactive Peptides Derived from Food Proteins across the Intestinal Epithelial Membrane: A Review. Trends Food Sci. Technol..

[5] Bohn T., Carriere F., Day L., Deglaire A., Egger L., Freitas D., Golding M., Le Feunteun S., Macierzanka A., Menard O. (2018). Correlation between *in Vitro* and *in Vivo* Data on Food Digestion. What Can We Predict with Static *in Vitro* Digestion Models?. Crit. Rev. Food Sci. Nutr..

[6] Baptista D.P., Gigante M.L. (2021). Bioactive Peptides in Ripened Cheeses: Release during Technological Processes and Resistance to the Gastrointestinal Tract. J. Sci. Food Agric..

[7] Shivanna S.K., Nataraj B.H. (2020). Revisiting Therapeutic and Toxicological Fingerprints of Milk-Derived Bioactive Peptides: An Overview. Food Biosci..

[8] Pihlanto-Leppälä A. (2000). Bioactive Peptides Derived from Bovine Whey Proteins: Opioid and Ace-Inhibitory Peptides. Trends Food Sci. Technol..

[9] Wieczorek Z., Zimecki M., Janusz M., Staroscik K., Lisowski J. (1979). Proline-Rich Polypeptide from Ovine Colostrum—Its Effect on Skin Permeability and on the Immune-Response. Immunology.

[10] Gill H.S., Doull F., Rutherfurd K.J., Cross M.L. (2000). Immunoregulatory Peptides in Bovine Milk. Br. J. Nutr..

[11] Jaziri M., Migliore-Samour D., Casabianca-Pignède M.R., Keddad K., Morgat J.L., Jollès P. (1992). Specific Binding Sites on Human Phagocytic Blood Cells for Gly-Leu-Phe and Val-Glu-Pro-Ile-Pro-Tyr, Immunostimulating Peptides from Human Milk Proteins. Biochim. Biophys. Acta (BBA)/Protein Struct. Mol..

[12] Parma J., Duprez L., Sande J.V., Cochaux P., Gervy C., Mockel J., Dumont J., Vassart G. (1993). Somatic Mutations in the Thyrotropin Receptor Gene Cause Hyperfunctioning Thyroid Adenomas. Nature.

[13] Miguel M., Contreras M.M., Recio I., Aleixandre A. (2009). ACE-Inhibitory and Antihypertensive Properties of a Bovine Casein Hydrolysate. Food Chem..

[14] Sorrenti V., Ali S., Mancin L., Davinelli S., Paoli A., Scapagnini G. (2020). Cocoa Polyphenols and Gut Microbiota Interplay: Bioavailability, Prebiotic Effect, and Impact on Human Health. Nutrients.

[15] Darling N.J., Mobbs C.L., González-Hau A.L., Freer M., Przyborski S. (2020). Bioengineering Novel *in Vitro* Co-Culture Models That Represent the Human Intestinal Mucosa with Improved Caco-2 Structure and Barrier Function. Front. Bioeng. Biotechnol..

[16] Boim A.G.F., Wragg J., Canniatti-Brazaca S.G., Alleoni L.R.F. (2020). Human Intestinal Caco-2 Cell Line *in Vitro* Assay to Evaluate the Absorption of Cd, Cu, Mn and Zn from Urban Environmental Matrices. Environ. Geochem. Health.

[17] Wang B., Xie N., Li B. (2019). Influence of Peptide Characteristics on Their Stability, Intestinal Transport, and *in Vitro* Bioavailability: A Review. J. Food Biochem..

[18] Le Roux L., Chacon R., Dupont D., Jeantet R., Deglaire A., Nau F. (2020). *In Vitro* Static Digestion Reveals How Plant Proteins Modulate Model Infant Formula Digestibility. Food Res. Int..

[19] Bagul M., Kakumanu S., Wilson T.A. (2015). Crude Garlic Extract Inhibits Cell Proliferation and Induces Cell Cycle Arrest and Apoptosis of Cancer Cells *in Vitro*. J. Med. Food.

[20] Gallego M., Grootaert C., Mora L., Aristoy M.C., Van Camp J., Toldrá F. (2016). Transepithelial Transport of Dry-Cured Ham Peptides with ACE Inhibitory Activity through a Caco-Cell Monolayer. J. Funct. Foods.

[21] Cieza R.J., Cao A.T., Cong Y., Torres A.G. (2012). Immunomodulation for Gastrointestinal Infections. Expert Rev. Anti. Infect. Ther..

[22] Hadi S., Akbar A., Chobert J., Haertle T. (2008). Kinetic Characterization of Hydrolysis of Camel and Bovine Milk Proteins by Pancreatic Enzymes. Int. Dairy J..

[23] Potolicchio I., Santambrogio L., Strominger J.L. (2003). Molecular Interaction and Enzymatic Activity of Macrophage Migration Inhibitory Factor with Immunorelevant Peptides. J. Biol. Chem..

[24] Lai Y., Gallo R.L. (2009). AMPed up Immunity: How Antimicrobial Peptides Have Multiple Roles in Immune Defense. Trends Immunol..

[25] Chen Y., Xue F., Xia G., Zhao Z., Chen C., Li Y., Zhang Y. (2019). Transepithelial Transport Mechanisms of 7,8-Dihydroxyflavone, a Small Molecular TrkB Receptor Agonist, in Human Intestinal Caco-2 Cells. Food Funct..

[26] Boyer J., Brown D., Liu R.H. (2005). *In Vitro* Digestion and Lactase Treatment Influence Uptake of Quercetin and Quercetin Glucoside by the Caco-2 Cell Monolayer. Nutr. J..

[27] Yin N., Cai X., Du H., Zhang Z., Li Z., Chen X., Sun G., Cui Y. (2017). *In Vitro* Study of Soil Arsenic Release by Human Gut Microbiota and Its Intestinal Absorption by Caco-2 Cells. Chemosphere.

[28] Wang Q., Qiao X., Qian Y., Li Z.-w., Tzeng Y.-m., Zhou D.-m., Guo D.-a., Ye M. (2015). Intestinal Absorption of Ergostane and Lanostane Triterpenoids from Antrodia Cinnamomea Using Caco-2 Cell Monolayer Model. Nat. Products Bioprospect..

[29] Brandsch M., Knütter I., Bosse-Doenecke E. (2010). Pharmaceutical and Pharmacological Importance of Peptide Transporters. J. Pharm. Pharmacol..

[30] Stroever S., Hayes K., Hice J.L., Baldauf G., Woodard C., Martin R. (2015). Construction and Implementation of an Operating Room Management Plan for the Prevention of Perioperative Hypothermia. Am. J. Infect. Control.

[31] Pappenheimer J.R., Dahl C.E., Karnovsky M.L., Maggio J.E. (1994). Intestinal Absorption and Excretion of Octapeptides Composed of D Amino Acids. Proc. Natl. Acad. Sci. USA.

[32] Adson A., Raub T.J., Burton P.S., Barsuhn C.L., Hilgers A.R., Audus K.L., Ho N.F.H. (1994). Quantitative Approaches to Delineate Paracellular Diffusion in Cultured Epithelial-Cell Monolayers. J. Pharm. Sci..

